# The Neuroprotective Effects of Astaxanthin: Therapeutic Targets and Clinical Perspective

**DOI:** 10.3390/molecules24142640

**Published:** 2019-07-20

**Authors:** Sajad Fakhri, Ina Yosifova Aneva, Mohammad Hosein Farzaei, Eduardo Sobarzo-Sánchez

**Affiliations:** 1 Pharmaceutical Sciences Research Center, Health Institute, Kermanshah University of Medical Sciences, Kermanshah 6718874414, Iran; 2 Institute of Biodiversity and Ecosystem Research, Bulgarian Academy of Sciences, 1113 Sofia, Bulgaria; 3 Laboratory of Pharmaceutical Chemistry, Department of Organic Chemistry, Faculty of Pharmacy, University of Santiago de Compostela, 15782 Santiago de Compostela, Spain; 4 Instituto de Investigación e Innovación en Salud, Facultad de Ciencias de la Salud, Universidad Central de Chile, Santiago 8330507, Chile

**Keywords:** neurodegenerative diseases, astaxanthin, pharmacology, neuroprotective agent, oxidative stress, neuroinflammation, apoptosis, drug delivery system

## Abstract

As the leading causes of human disability and mortality, neurological diseases affect millions of people worldwide and are on the rise. Although the general roles of several signaling pathways in the pathogenesis of neurodegenerative disorders have so far been identified, the exact pathophysiology of neuronal disorders and their effective treatments have not yet been precisely elucidated. This requires multi-target treatments, which should simultaneously attenuate neuronal inflammation, oxidative stress, and apoptosis. In this regard, astaxanthin (AST) has gained growing interest as a multi-target pharmacological agent against neurological disorders including Parkinson’s disease (PD), Alzheimer’s disease (AD), brain and spinal cord injuries, neuropathic pain (NP), aging, depression, and autism. The present review highlights the neuroprotective effects of AST mainly based on its anti-inflammatory, antioxidative, and anti-apoptotic properties that underlies its pharmacological mechanisms of action to tackle neurodegeneration. The need to develop novel AST delivery systems, including nanoformulations, targeted therapy, and beyond, is also considered.

## 1. Introduction

Neurodegenerative disorder, as a common cause of human disability and death, is a term referring to progressive, symmetric, and selective loss of sensory, motor, and cognitive neuronal structure/function leading to neuronal cell death [1]. The death of neurons underlies the symptoms of several chronic or acute neurological disorders including Parkinson’s disease (PD), Alzheimer’s disease (AD), and brain or spinal cord injuries [2]. Neuronal cell death also affects depression, neuropathic pain (NP), aging, and autism as other neurological disorders [3]. 

Several causative factors are behind the etiology of neuronal disorders such as oxidative stress, inflammation, and apoptosis, as the main pathological pathways, and they play destructive roles in neuronal cell death and neurodegenerative processes [4]. Microglia activation and cytokines/chemokines release of the inflammatory pathways [5], as well as reactive oxygen species (ROS) and mitochondrial damages in the oxidative stress pathway [6], have destructive effects on neurodegenerative processes, which finally lead to cell death [4]. 

In spite of many developments in the field of clinical healthcare, neuroprotective therapies of neurodegeneration and neuronal death-related disorders have still remained as clinical challenges with no effective solution. Therefore, the need to develop novel multi-target therapeutics is felt to regulate more involving the signaling pathways and is thought to improve the life quality of individuals with neurodegenerative diseases. 

Carotenoids are red-orange lipophilic pigments found in nature [7,8] with protective effects for human health. Thanks to their potential biological activities and health benefits, carotenoids have been receiving growing attention [4,9]. Several reports have attributed positive effects to their antioxidant activities [10,11]. Being the strongest antioxidant between the carotenoids [9], Astaxanthin (AST) is a lipid-soluble keto-carotenoid, belonging to xanthophylls, which has gained attention in experimental methods due to its neuroprotective features [12,13]. 

**Figure 1:**
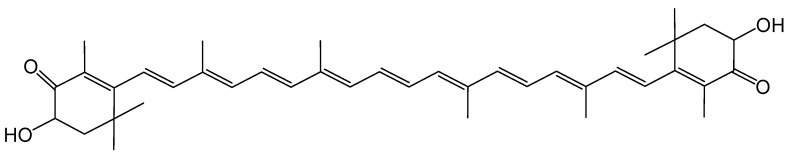
Chemical structure of Astaxanthin (AST).

AST can be isolated mostly from microalgae *Haematococcus pluvialis*. However, shrimp, asteroidean, algae, lobster, crustacean, krill, trout, red sea bream, and salmon as marine animals and seafood are considered as other sources of extraction [4]. AST is chemically known as 3,3′-dihydroxy-β, β′-carotene-4,4′-dione (Figure 1), with the molecular formula of C_40_H_52_O_4_ [14]. It possesses a linear polar-nonpolar-polar structure with keto- and hydroxyl moieties at polar ends and conjugated carbon-carbon double bonds at a non-polar middle part, which allows it to fit specifically into the same span of cell membranes and pass through blood-brain barrier (BBB) [15]. 

It has been shown that AST can block oxidative stress, inflammation, and apoptosis as the key pathways of neurodegeneration [4]. In the inflammatory pathway, AST blocks the macrophage migration inhibitory factor (MIF) as an up-stream cytokine, N-methyl-D-aspartate (NMDA) receptor 2B (NR2B) [12,13], and IκB kinase β (Ikk β). Thus, it inhibits the release of interleukins (ILs), tumor necrosis factor alpha (TNF-α), intercellular adhesion molecule 1 (ICAM1), and monocyte chemoattractant protein-1 (MCP-1) [4]. To tackle the oxidative stress, AST inhibits phosphorylated extracellular regulated protein kinase/extracellular regulated protein kinase ratio (p-ERK/ERK) [12,13], activates Nrf2/antioxidant response elements (Nrf2/ARE), and increases the release of heme oxygenase-1 (HO-1), glutathione S- transferase-α1 (GST-α1), and NAD(P)H quinine oxidoreductase-1 (NQO-1) [4,16]. Indeed, as the major signaling pathway against oxidative stress, Kelch-like ECH-associated protein 1 (Keap1)-Nrf2-ARE plays a key role in the cellular antioxidant response. In the non-stressed situation, Keap1 degrades the Nrf2 protein in the cytoplasm through the proteasome. Upon the oxidative stress, Nrf2 is rapidly degraded by proteasomes via the interaction with Keap1. This modification leads to the cytoplasmic accumulation and the nucleus translocation of newly synthesized Nrf2 in order to bind to the ARE [17,18]. Nrf2-ARE complex, in turn, attenuates the expression of NAD(P)H quinone oxidoreductase-1, superoxide dismutase (SOD), HO-1, and other regulatory enzymes to activate the defense system [18,19]. While glutathione reductase and thioredoxin reductase protect the complexes stability of Keap1-Nrf2 as well as IκB-NF-κB, cytosolic H_2_O_2_ cause dissociation of the complexes and allows the nuclear transportation of NF-κB and Nrf2 [20,21]. In this line, the Nrf2-ARE pathway is believed to decrease ROS concentrations in order to keep a balance between ROS and the antioxidant potentials [22,23]. AST also acts against apoptosis by blocking p-ERK/ERK [12,13], cytochrome c, caspase3,9, and the Bax/Bcl2 ratio [4,24]. 

Nevertheless, considering the unsaturated structure of AST, it is highly susceptible to light, oxygen, and heat stress degradation. In addition, the poor water solubility and bioavailability of AST [25,26,27,28] limit its efficacy in vivo [29,30]. So, investigating novel AST delivery system is necessary in order to solve these drawbacks. 

To the best of our knowledge, this is the first review regarding the role of each signaling pathway in the pathogenesis of neurodegenerative disorders, including PD, AD, NP, depression, brain and spinal cord injuries, aging, and autism, and it is the first review regarding the auspicious effects of AST against neurodegeneration pathways as well as clarifying novel AST delivery systems, all together. 

## 2. AST and Neurodegenerative Diseases

### 2.1. AST and Parkinson’s Disease (PD)

PD is an age-related disorder and the second most common cause of neurodegenerative disorders with a prevalence of 0.1–0.2% worldwide and 3% in people older than 80 years old [31,32]. PD is characterized by midbrain dopaminergic neurons lost, aggregation of α-synuclein called Lewy bodies, and destruction of non-dopaminergic pathways leading to motor and non-motor dysfunction [33,34]. 

Due to their low efficacy and undesirable adverse effects, conventional treatments of PD are quite challenging. Hence, discovering an effective and safe novel multi-target agent to combat PD is of great importance. 

Neuroinflammation and oxidative stress have a major contribution in the pathogenesis of PD [35,36]. The inhibition of the mediators of these pathways plays an important role in preventing the disease progression, which is what AST does as a multi-target drug. In a rat model of homocysteine (Hcy)-induced hippocampal neurotoxicity and apoptosis, AST regulated ROS-mediated oxidative damage and mitochondrial dysfunction. AST also attenuated PI3K/AKT and mitogen-activated protein kinase (MAPK) pathways and thus, was used to tackle these neurological disorders, such as PD [37]. AST acted through the SP1/NR1 and HO-1/NOX2 axis to inhibit MPP+ induced oxidative stress in PC12 cells [38,39]. MPTP (1-methyl-4-phenyl-1,2,3,6-tetrahydropyridine) as a progressive cause of PD in experimental models [40] affected the hydroxylase activity of tyrosine as an enzyme involved in dopamine biosynthesis [41]. 

AST conserved substantia nigra from MPTP-induced dopaminergic neuronal loss in aged and young mice, but it was not able to protect against the loss of tyrosine hydroxylase induced by MPTP in aged mice. Therefore, Grimmig et al. considered aging as a critical factor in finding novel therapies for PD [42]. 

In an in vitro study, Lee and colleagues reported that AST ameliorated MPP+-induced production of ROS in SH-SY5Y human neuroblastoma cells. This effect may be attributed to a decrease in α-synuclein, caspase-3, and the Bax/Bcl-2 ratio and the increase of SOD, catalase, and tyrosine hydroxylase [43]. 

Moreover, Liu et al. indicated that AST pretreatment inhibited 6-hydroxydopamine (6-OHDA) or DHA hydroperoxide (DHA-OOH)-induced apoptosis, intracellular ROS generation, and mitochondrial dysfunctions in dopaminergic SH-SY5Y cells through its antioxidant potential and mitochondria protection [44]. Also, AST inhibited 6-OHDA-induced apoptosis and mitochondrial dysfunction via blocking the phosphorylation of p38 MAPK and reducing caspase 3/9 and poly(ADP-ribose) polymerase [45]. 

### 2.2. AST and Alzheimer’s Disease (AD), Cognition, and Memory

The brain has rich irrigation with blood vessels, very high oxygen consumption, and lower antioxidant capability, which it is very susceptible to oxidative damage [46]. AD is an age-related neurodegenerative disease, which is characterized by the overproduction and deposition of beta-amyloid peptide (Aβ) plaques and intracellular neurofibrillary tangles, and by a loss of neurons in the brain [47,48]. One of the main reasons for the development and progression of AD is the oxidative stress [49,50]. AST is one of the few compounds that can cross the blood-brain barrier (BBB) in mammals, and going beyond this barrier could increase their antioxidant properties. The molecular mechanisms still are not elucidated but there are many types of research focused on neuronal apoptosis. Shen et al. reported that AST reduced ischemia-related injury in brain tissue, mainly through the inhibition of oxidative stress. It also protected neuroblastoma cells against Aβ-induced oxidative cell death through induction of the antioxidant enzyme HO-1 expression [51]. Later, Wen et al. investigated the neuroprotective effects of AST on glutamate-induced oxidative ex situ toxicity in a mouse hippocampal HT22 cells through Nrf2-dependent HO-1 expression [52]. The results indicated that AST is a promising biologically active compound for the treatment of neurodegenerative disorders such as AD. The amount of glutathione in the plasma has a correlation with the severity of cognitive dysfunction in AD patients [53]. Another study demonstrated that AST apparently showed a protective effect on L-glutamate-induced PC12 cell death mainly through the Bcl-2/Bax signaling pathway and, therefore, it could be considered a promising agent as prophylactic or remediation against neuronal disorders [54]. An experiment with double transgenic mice administrated with AST and its synthesized variant docosahexaenoic acid-acylated AST diesters (AST-DHA) for 2 months suggested that AST-DHA might be a potential therapeutic agent for AD. In the study, Radial 8-Arm Maze Test, Water Maze Test, Determination of Aβ Concentration, and western blot analysis was carried out [55]. 

Many studies aimed at exploring the relationship between diet and their effects on cognitive ability [56,57]. Hussein et al. reported the neuroprotective actions of AST and its high potential in human health and nutrition [58]. The contribution of fish oil in the process is an important and significant step in the protection of the nervous system and especially of the brain [59,60]. A protective function of AST in microcirculation and mitochondrial functions was demonstrated [15], which confirms its potential efficacy in several neurodegenerative diseases [61]. 

### 2.3. AST and Neuropathic Pain (NP)

NP is caused by a disease or a lesion in the somatosensory nervous system [62], with an estimated cost of 40 billion dollars per year in the U.S. [63]. Several destructive signaling pathways and mechanisms are involved in NP, mostly including neuromodulators (glutamate, and especially NR2B, gamma-aminobutyric acid (GABA), serotonergic, and noradrenergic) and inflammatory agents (cytokines, prostaglandins, and reactive oxygen species) [64], which affect microglia and astrocytes activation, ion currents, and neuronal firing [65,66], as well as apoptosis [67]. 

Antidepressants and anticonvulsants are among the primary clinical alternatives for the management of NP [68]. Nevertheless, investigating novel multi-target pharmacological therapies for NP, which simultaneously target multiple destructive mediators with acceptable efficacy and safety, is of great importance. 

As Sharma et al. reported, AST attenuated biochemical and behavioral alterations using in vivo and in vitro models of NP. They found that AST decreased astrocytic activation. Thereby, glial fibrillary acidic protein (GFAP) afforded suppression and reduced oxido-nitrosative stress in vitro. Also, AST antagonized NR2B in silico and reduced thermal and mechanical allodynia in a rat model of chronic constriction injury (CCI)-induced NP [69]. In the same in vivo model of NP, AST prevented the increase in IL-6, IL-1β, and TNF-α in the spinal cord and hippocampus of mice [70]. Fakhri and colleagues confirmed the neurotoxic role of NR2B as a glutamate-gated channel, as well as the inhibitory effects of AST on NR2B and the glutamate-initiated signaling pathways in a rat model of compression spinal cord injury (SCI). It was also found that, besides improving neuronal damages, AST down-regulated TNF-α and p-p38MAPK, through which neuroinflammation and mechanical allodynia was inhibited [13]. We also confirmed the neuroprotective effects of AST in reducing the cold allodynia passed through the inhibition of p-ERK/ERK and the activation of p-AKT/AKT [12]. In a carrageenan-induced mice model of pain and paw edema, AST decreased thermal and mechanical allodynia, as well as the lipid peroxidation and myeloperoxidase enzyme in the paw [71]. 

Altogether, AST is introduced as an effective drug to combat NP. Additionally, since patients with chronic NP are at high risk of co-morbid depression [70], the need to investigate the antidepression effects of AST is greatly raised. 

### 2.4. AST and Depression

Depression is a common and complicated psychological condition for human health. As a major cause of mortality and morbidity, depression is predicted to be the main pathogeny of disability by 2030 [72]. Since the complex pathophysiology of depression is not yet completely known, the most suitable treatments are yet to be clarified [73]. In the treatment of depression, monoamine regulation still plays a crucial role and brings up tricyclic antidepressants (TCAs), selective serotonin reuptake inhibitors (SSRIs), monoamine oxidase inhibitors (MAOIs), serotonin-norepinephrine reuptake inhibitors (SNRIs), and selective–norepinephrine reuptake inhibitors (SNERIs) [74,75], although most of them have low efficacy with potential adverse effects [76]. On the other hand, according to recent evidence, there is a close connection between depression and oxidative stress/inflammation, as non-monoaminergic pathways are involved in depression, which are now areas of active investigation. Besides, destructive intracellular pathways of oxidative stress and inflammation also underlie the etiology of depression and anxiety [77,78,79]. Altogether, investigating novel multi-target therapeutic agents for depression with acceptable safety and efficacy is still a medical need. 

Several studies have reported the antidepressant-like effects of AST in different experimental models. As reported by Zhou and colleagues, AST prevented hyperglycemia-induced neuroinflammation contributing to depression. They also found that AST had an antidepressant-like effect by decreasing the level of IL-1β, IL-6, cyclooxygenase-2 (COX-2), cleaved caspase-3, and GFAP, and protecting neurons in the amygdala, hypothalamus, and hippocampus of mice [80]. In this context, Jiang et al. found that *trans*-AST ameliorated lipopolysaccharide (LPS)-induced depressive-like behaviors through the down-regulation of TNF-α, IL-6, and IL-1β and by antagonizing inducible nitric oxide synthase (iNOS), neuronal nitric oxide synthase (nNOS), and COX-2 expression in mice [81]. Chronic treatment with *trans*-AST also prevented co-morbid depression in mice, owing to their potent anti-inflammatory effects and involvement in the serotonergic pathway [70]. In this sense, the involvement of the serotonergic pathway in the pathogenesis of depression and related inhibitory effect of *trans*-AST were raised again [82]. 

In a rat model of depression, AST reverted the antagonistic and impairing effects of ethanol on cortical spreading depression in a dose-dependent [83], but not age-dependent [84], manner, which was attributed to the antioxidant effects of AST [83]. Moreover, Qiao et al. used a mice model of omethoate-induced depression and found that a combination therapy of AST and lithium chloride efficiently attenuated depressive-like behavior through the Akt/GSK3β/CREB signaling pathway [85]. 

As another crucial non-monoaminergic mechanism, the NMDA receptor and the glutamatergic pathway play destructive roles in the pathogenesis of depression. A type-specific NMDA receptor antagonist could offer more efficacy and fewer complications related to broader NMDA receptor blockers. Since AST blocks NR2B efficiently [13], it can be suggested as a strong anti-depressant drug. 

### 2.5. AST and Central Nervous System (Brain/Spinal Cord) Injuries

Central nervous system (CNS) injuries, including brain and spinal cord injury, affect millions of individuals worldwide [86,87]. As complex processes of primary and secondary phases, CNS injury phases initiate temporary or permanent neuronal damages. Following the mechanical injury, the primary phase is characterized by direct death of cells followed by the secondary phase, consisting of inflammatory, oxidative, apoptotic, and other molecular pathways that cause further edema and damages to neuronal cells by inciting a breach in the BBB [88,89,90]. There are several destructive mediators that can be targeted by neuroprotective agents to prevent CNS injuries. However, there are still no sufficient data available regarding the improvement of post-CNS injuries. 

In the context of brain injury, AST treatment attenuated early brain injury (EBI) after subarachnoidal hemorrhage (SAH) by reducing the brain edema, BBB disruption, and caspase3. This neuroprotective effect of AST has been attributed to its strong antioxidant property by decreasing malondialdehyde (MDA) and increasing glutathione (GSH) and SOD in rodent models [90]. In an in vivo study, AST significantly prevented H_2_O_2_-induced apoptosis, improved neurological deficit, and diminished the infarct volume, as well [91]. Also, AST suppressed oxygen-glucose deprivation (OGD)-induced oxidative stress by upregulating the protein expression of HO-1, Hsp32, and Hsp90 in SH-SY5Y cells. Thus, it is confirmed that the neuroprotective effects of AST in CNS damages are also related to its antioxidant effects [92]. 

Following EBI in SAH model, AST positively attenuated the cortical expression of NAD (P) H: quinone oxidoreductase 1 (NQO-1), HO-1, and glutathione S-transferase-α1 (GST-α1) through the antioxidant pathway named Nrf2-ARE at both mRNA and protein levels. Additionally, AST ameliorated BBB disruption, brain edema, apoptosis, and neurological dysfunction in this context [93]. 

Considering that apoptosis plays a crucial role in the pathogenesis of EBI, AST considerably increased the phosphorylation of Akt and Bad levels, which led to a reduction in apoptosis and caspase-3 levels following SAH [94]. AST ameliorated mitochondrial membrane potential, cerebral vasospasm, and mitochondria-associated neuronal apoptosis by reducing caspase-3, the Bax/Bcl-2 ratio, and cytochrome c in the prefrontal cortex post-SAH [95]. 

Zhang et al. found that treatment with AST prevented SAH injury by inhibiting the toll-like receptor 4 signaling pathway and increasing sirtuin 1 and the subsequent inflammatory response, both in vivo and in vitro [96]. This indicated the role of inflammation, besides oxidative stress and apoptosis, in CNS injuries. In a rat model of SAH, AST down-regulated matrix metallopeptidases-9 (MMP-9) which was attributed to a decrement in the level of infiltrating neutrophils, activated microglia, TNF-α, and IL-1β [97]. 

Recent advancements have also clarified the contribution of Na^+^/K^+^/2Cl co-transporters (NKCCs) and aquaporins (AQPs) to brain edema during traumatic brain injury (TBI). Following TBI, AST attenuated AQP4/NKCC1-level in mice brain tissue [98]. According to Zhang and colleagues, AST down-regulated NKCC1 expression through the nuclear factor-kB (NF-κB) pathway, which mediates pro-inflammatory factors, and also protecting astrocytes against TBI [99]. 

In a rat model of SCI, Fakhri et al. reported that AST down-regulated NR2B, TNF-α, and p-p38MAPK, and it also preserved the tissue and neuronal damages and improved the sensory-motor function following a rat model of compression SCI [13]. We also found that AST increased and decreased the protein expression ratio of p-AKT/AKT and p-ERK/ERK, respectively, following SCI [12]. 

### 2.6. AST and Aging

The oxidative stress increases with aging and the brain become significantly more vulnerable to neurodegenerative disease [100,101]. The deficits in memory formation in older individuals caused by oxidative stress affect synaptic plasticity in neural networks in the hippocampus by protecting D-serine-dependent NMDA receptor activation. Hippocampal neurogeneration is associated with learning and memory processes [102]. Another study in mice showed that hippocampal oxidative stress is age dependent [103]. The reaction of young and aged animals to AST treatment on brain oxidative markers showed that there are no significant differences among them, and AST improves all types of oxidative markers in the six studied brain regions—namely the frontal cortex, striatum, parietal cortex, hypothalamus, hippocampus, and cerebellum [104]. The effects of AST on the aging female and male rat brains have been analyzed and the results obtained showed the gender-related differences [105]. 

Researchers studied the inhibition of oxidative injury of biological membranes by AST and found that its efficacy is higher than vitamin E, which is associated with the mitochondrial theory of aging [106]. This property highlights its unique potential to combat aging [4,107]. Hussein et al. marked the properties of AST for the prevention of age-related macular degeneration [58], and Wu et al. reported its effect on inhibition of apoptosis and alleviation of injury in the brains of aging rats [108]. 

Topically applied, AST affected aging skin with a visible wrinkle reduction. There are some clinical studies supporting this statement [109]. Yamashita found that the combined use of a dietary supplement containing AST and tocotrienol from palm oil resulted in a significant reduction of fine wrinkles [110]. On the other hand, a single-blind placebo-controlled study showed that dietary supplement containing only AST with a dose of 4 mg per day led to significant improvements in human skin characterized by elasticity during a dermatologist’s visual assessment [109]. The combination of oral supplementation with topical application resulted in significant improvements in skin wrinkles, age spot size, elasticity, skin texture, moisture, the content of the corneocyte layer, and the corneocyte condition [111]. 

### 2.7. AST and Autism

As a neurodegenerative disease with an increasing prevalence, autism is characterized by impairments in communication, social interaction, behavior, and regular activities [112,113,114]. Age-related and progressive neuronal pathology and neuronal loss in cerebellar Purkinje [115] or amygdala [116] cells in individuals with autism [117] are all representative of a neurodegenerative process [118,119,120,121]. There is also a close link between autism and the release of proinflammatory cytokines [122,123]. Thus, AST is a multi-target anti-neuroinflammatory drug that can be used against autism. 

Besides, given the importance of oxidative stress in the pathogenesis of autism [124,125,126,127], AST as the strongest antioxidant among the carotenoid pigments [9] can be used to combat autism, as Ornoy et al. also reported [128]. On the other hand, the plasma concentrations of exogenous antioxidants, such as carotenoids, are insufficient in autistic adolescents and children, which confirms the role of oxidative stress in the pathogenesis of autism and the inhibitory effects of carotenoids [129]. 

AST increased the paw withdrawal latency, evaluated by a hot plate test, and improved the behavioral disorders, assessed by the social interaction and open field tests, in a mice model of pre-natally valproic acid-induced autism. In the same context, AST treatment also reduced oxidative stress through the reduction of nitric oxide and lipid peroxidation and the increment of catalase activity [104]. Frutos et al. reported that due to their anti-oxidative and anti-inflammatory properties, carotenoids are introduced as potential foods against autism [130]. 

Considering the role of oxidative stress and neuroinflammation signaling mediators in the pathogenesis of autism, each of these mediators can be a target to combat autism. Even though there is not enough evidence regarding the effects of AST on autism, it can be suggested as an auspicious neuroprotective agent against autism due to its inhibitory role on neuroinflammation and oxidative stress. 

**Figure 2:**
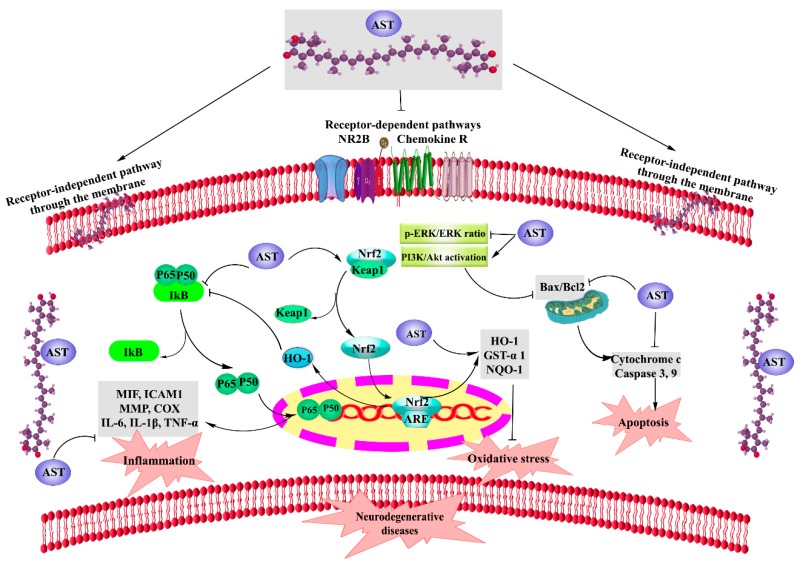
Neuroprotective mechanisms of AST. NR2B: N-methyl-D-aspartate (NMDA) receptor 2B, Chemokine R: Chemokine receptor, HO-1: heme oxygenase-1, GST-α1: glutathione-s-transferase-α1, NQO-1: NAD(P)H quinone oxidoreductase-1, MDA: malondialdehyde, ARE: antioxidant response elements, PI3K/AKT: phosphoinositide 3-Kinase/AKT, p-ERK/ERK: phosphorylated extracellular regulated protein kinase/extracellular regulated protein kinase ratio, MIF: macrophage migration inhibitory factor, ICAM1: Intercellular adhesion molecule 1, MMP: Matrix metallopeptidase, TNF-α: Tumor necrosis factor alpha, NOS: Nitric oxide synthase, COX: Cyclooxygenase, IL: Interlukine, Bax/Bcl2: Bax/Bcl2 ratio, and AST: astaxanthin.

Figure 2 shows the neuroprotective mechanisms of AST for combating neurodegenerative diseases (Figure 2). 

## 3. AST Novel Delivery Systems: Nanoformulations, Targeted Therapy, and Beyond

As a division of nanotechnology, nanomedicine uses biocompatible and biodegradable nanoaggregates and submicron-sized nanoparticles to target pharmacokinetics, administration routes, and bioavailability of drugs in medicine [131]. 

AST is a lipid-soluble carotenoid with low bioavailability that is partly absorbed by intestinal cells. It displays poor dispersibility and water solubility in aqueous solutions and is susceptible to light, oxygen, and heat stress degradation [25,26,27,28]. In spite of the strong in vitro neuroprotective effects of AST, the lack of an appropriate AST-delivery system to pass through BBB fails to show the same responses in vivo. Such characteristics of AST have prevented it from being widely used in biomedical or pharmaceutical applications. On the other hand, various approaches have so far been applied to formulate AST into a novel drug delivery system in order to increase its stability, solubility, and bioavailability, and to prolong its shelf life [29,30]. In addition to low bioavailability and lack of an appropriate drug delivery system, high thermolability, instability, and lipophilicity of AST have caused its antioxidant efficacy to be failed in clinical trials. Thus, developing an appropriate drug delivery system capable of overcoming these limitations is of great necessity. However, there are promising ways to overcome these limitations and to improve the performance of pharmaceuticals to prepare a suitable nanoformulation of AST. In this regard, different formulations of AST-loaded lipid-based carriers (LBCs), polymeric systems, and inclusion complexes have been provided to potentiate its effect [132]. 

As an auspicious delivery system, LBCs offer to enhance the stability and bioavailability of active pharmaceutical ingredients (API) while allowing a controlled release [132,133] achieved by a surface charge or adsorption of a layer of polymer or surfactant. LBCs consist of oil in water (O/W) nano/microemulsions, oil-loaded solid lipid nanoparticles (SLN), nanostructured lipid carriers (NLC), and micelles [134]. 

As dispersions of small spheroid within an aqueous medium, O/W microemulsions are thermodynamically stable, while nanoemulsions are unstable colloids [134,135]. The bioavailability of AST nanoemulsions has been reported to increase through the elevation of its oxidative and physical stability [136,137]. SLNs are mixtures of O/W nano/microemulsions with the high-ordered inner crystalline structure of lipid phase. SLN-AST is shown to possess unique properties such as large surface area, high drug loading, small size, and a wide spectrum of biodistribution, which, in return, realizes the goals of site-specific and controlled drug delivery as a promising strategy for efficient delivery of hydrophobic drugs. It offers more potential neuronal applications for passing through BBB to protect the brain from oxidative stress and to provide valuable support for brain health [138,139]. As reported by Bhatt et al., SLN-AST was shown to be an effective treatment to tackle oxidative stress-induced neurodegeneration in pheochromocytoma-12 cell line [139]. AST nanoemulsion also plays a momentous role in the stimulation of oxidative stress and mitochondrial-mediated apoptosis in cancer cells [140]. So, mitochondrial-targeted therapy via novel delivery systems could potentiate the effect of AST. Since drug-loaded nanoemulsion is a lipophilic molecule, it is likely to be localized to the membrane, followed by the mitochondria and nucleus [141]. Such drug delivery systems are implemented to selectively target mitochondria, and the targeted therapy was confirmed by apoptotic changes, mitochondrial membrane potential, and intracellular changes of membrane ROS [140]. 

To compensate for the low bioavailability and capacity of drug loading and release in SLN, NLCs were developed with a less-ordered inner crystalline structure allowing higher bioavailability and loadings of the lipophilic API with easier scale-up [142,143,144,145]. Indeed, NLCs, as more superior options than other colloidal carriers, present a remarkable capability to preserve, stabilize, and amplify the antioxidant capacity of AST [132,133,146,147]. Rodriguez-Ruiz et al. reported that ASTCO2-NLCs could be potentially introduced as excellent candidates for developing new platforms applications for medical devices, as well as antioxidant delivery systems for nutraceuticals [146]. Tamjidi et al. showed no drastic effects of ionic strength, heat, pH, and simulated gastric juice on the chemical stability of AST-loaded NLCs [148]. Several other studies also suggested NLCs as a successful AST-delivery system [29,149]. Although NLCs improve the functionality of AST from different aspects, the key role of emulsifiers in the system composition cannot be neglected [150,151], indicating the strong effect of the emulsifier on the bioavailability of AST-loaded NLCs [152]. 

Along with SLNs and NLCs, nanoliposomes as nano-scaled colloid systems made from the dispersion of amphiphilic lipids in aqueous solvents are considered to be other successful lipid base carriers in delivery systems [153]. Nanoliposomes benefit from such characteristics as higher membrane penetration and bioavailability, as well as superior potentiality to be used for specific drug targeting systems [154]. Pan et al. revealed that AST-loaded nanoliposomes increased the membrane micropolarity and decreased the membrane fluidity in order to attenuate the membrane structural properties [153]. AST-loaded liposomes functioned as a promising drug delivery system to treat hepatotoxicity [155] with increased bioavailability [155,156]. According to Peng et al., liposome encapsulation also elevated the stability, transportability, and antioxidation properties of AST [156]. The potential of liposomal encapsulation to enhance the radical scavenging effects of AST has been shown by other studies as well [157,158,159]. 

Polymeric systems have also been recently used as other novel delivery systems to enhance the solubility, protection of the biological activity, physicochemical stability, and the antioxidant effects of AST, and also to control its release. Due to their biodegradability and biocompatibility properties, chitosan [160,161] and alginate [134,162,163,164], as natural polysaccharides, are used in polymeric delivery systems of AST microencapsulation, leading to higher cell uptake and antioxidant effects of AST. The absorption profile of polymeric nanosystems of AST is affected by its shape, particle size, and surface properties, which could be used to handle the release of AST [164]. 

**Figure 3:**
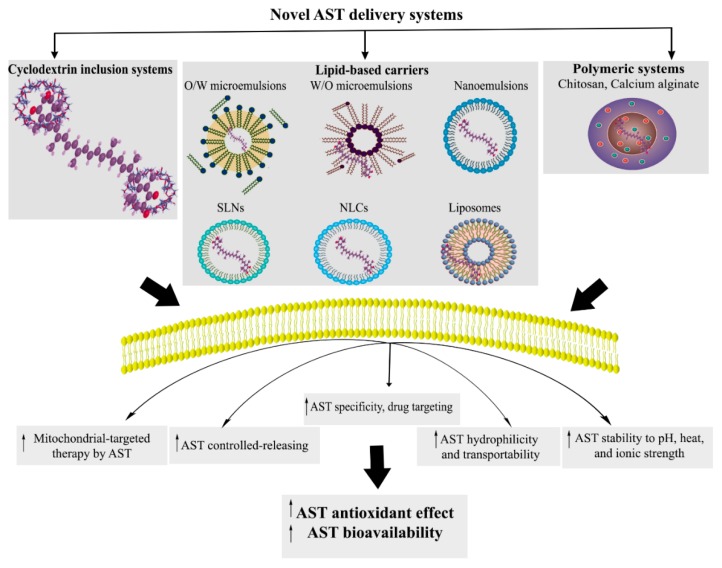
Novel AST delivery systems. O/W: oil in water, W/O: water in oil, SLNs: solid lipid nanoparticles, NLCs: nanostructured lipid carriers, and AST: astaxanthin.

Another upcoming method for AST delivery is to use the inclusion complex formation of cyclodextrin. It is a natural macrocyclic oligosaccharide with a hydrophilic outer surface and a lipophilic cavity used to enclose lipophilic molecules like AST [165]. The involved non-covalent guest–host associations result in the controlled release of the guest, increase of its hydrophilicity, stability against light, heat, and oxygen [166], and bioavailability [167], as well as an improvement in the antioxidation potential of the guest [168]. In several studies, cyclodextrin has shown the potential to increase the stability, hydrophilicity, bioaccessibility [169,170], and antioxidant activity of AST [168]. It is worth mentioning that cyclodextrin inclusion formation gives more hydrophilicity activity to AST, compared to NLCs and polymeric delivery systems. Figure 3 indicates novel AST delivery systems as well as their effects on AST properties (Figure 3). 

From the diagnostic point of view, AST nanoparticles could also be used for diagnostic purposes. Bharathiraja et al. reported the potential of AST gold nanoparticles in the photo-based diagnosis of cancer in the near infra-red range [171]. 

In general, the novel delivery systems of AST, including lipid carriers, mitochondrial targeted, polymeric, and cyclodextrin inclusion systems, could be considered as new ways to potentiate the effects of AST by improving its stability, hydrophilicity, and antioxidant capacity to be used in several diseases (i.e., neurodegenerative disorders). 

## 4. Conclusions and Future Perspective

AST is an oxy-carotenoid with potential effects on healthcare. Although most of the studies have considered the protective effects of AST on various diseases, recent studies have focused more on their neuroprotective effects. As a multi-target neuroprotective agent, AST affects multiple mechanisms of action to tackle complex pathophysiological mechanisms of neurodegenerative diseases, mainly based on its anti-inflammatory, antioxidant, and antiapoptotic effects. All neuroprotective pharmacological mechanisms of action of AST are highlighted in the current review. On the other hand, the lack of an appropriate drug delivery system of AST has caused its efficacy to be failed in clinical trials. So, investigating an appropriate AST delivery system in order to solve this drawback is of great importance. 

Such reports will provide novel applications of AST in the prevention, management, and treatment of neurodegenerative diseases, as well as investigating the most potential novel AST delivery system in clinical trials. Additional studies are needed to elucidate the precise pathophysiological pathways involved in neurodegeneration, and to clarify the potential neuroprotective effects of appropriate AST formulations on humans.
